# Association of the gene polymorphisms of *BMPR2, ACVRL1, SMAD9* and their interactions with the risk of essential hypertension in the Chinese Han population

**DOI:** 10.1042/BSR20181217

**Published:** 2019-01-25

**Authors:** Yunpeng Chen, Chenxi Ye, Jingwen Chen, Dongming Lin, Hao Wang, Shen Wang

**Affiliations:** 1 Department of Cardiology, the Affiliated Wenling Hospital of Wenzhou Medical University, the First People’s Hospital of Wenling, Taizhou 317500, China; 2 Department of Cardiology, the Second Affiliated Hospital of Zhejiang Chinese Medical University, Hangzhou 310005, China

**Keywords:** ACVRL1, BMPR2, essential hypertension, single nucleotide polymorphism, SMAD9

## Abstract

**Objective:** Genetic factors are involved in the occurrence, development, and progression of essential hypertension (EH). To study the association between single nucleotide polymorphisms (SNPs) of the rs6435156 and rs1048829 loci of the bone morphogenetic protein receptor type 2 (BMPR2) gene, the rs121909287 and rs121909284 loci of the activin receptor-like kinase 1 (ACVRL1) gene, and the rs397514716 and rs121918359 loci of the mothers against decapentaplegic homolog 9 (SMAD9) gene with the risk of EH in the Chinese Han population. **Materials and methods:** A total of 460 EH patients and 460 healthy controls were recruited for the study. Genomic DNA of white blood cells was extracted, and the genotypes were analyzed by Sanger sequencing after polymerase chain reaction amplification. Multi-factor dimensionality reduction (MDR) was used to analyze the effect of gene–environment interactions on EH risk. **Results:** The risk of EH increased in the *BMPR2* gene rs6435156 locus dominant model (adjusted odds ratio [OR] = 1.572, 95% confidence interval [CI]: 1.385–1.765, *P*<0.001) and recessive model (adjusted OR = 1.926, 95% CI: 1.693–2.067, *P*<0.001). The risk of EH increased in the rs1048829 recessive model (adjusted OR = 1.444, 95% CI: 1.142–1.696, *P*=0.003). The risk of EH increased in the recessive model of the *ACVRL1* gene rs121909287 locus (adjusted OR = 1.403, 95% CI: 1.101–1.660, *P*=0.008). The risk of EH increased in the *SMAD9* gene rs397514716 locus dominant model (adjusted OR = 1.370, 95% CI: 1.183–1.559, *P*<0.001) and recessive model (adjusted OR = 1.803, 95% CI: 1.470–1.983, *P*<0.001). The CG haplotype of the rs6435156 and rs1048829 loci of the *BMPR2* gene, the CC haplotype of the *ACVRL1* gene rs121909287 and rs121909284 loci, and the CC haplotype of the rs397514716 and rs121918359 loci of the *SMAD9* gene were factors that protect against EH, whereas the TT haplotype of the rs6435156 and rs1048829 loci in the *BMPR2* gene was a risk factor for EH. MDR analysis showed that the *BMPR2* gene rs6435156 locus TT genotype carriers, the *SMAD9* gene rs397514716 locus TT genotype carriers, and alcohol drinkers had the highest EH risk (OR = 4.523, 95% CI: 2.235–6.871, *P*<0.001). **Conclusion:** The SNPs of the rs6435156 and rs1048829 locus in the *BMPR2* gene, the rs121909287 loci in the *ACVRL1* gene, and the rs397514716 locus in the *SMAD9* gene were associated with a risk of EH in Han Chinese.

## Introduction

Hypertension is a major risk factor for cardiovascular disease-related death [1]. Hypertension is a polygenic genetic disease that its occurrence is determined by genetic and environmental factors. The drug treatment for hypertension reflects obvious individual differences [2], and with the development of pharmacogenomics, determining the type and dosage of drugs on the basis of individual genotype has become an important means of disease treatment.

The human *BMPR2* gene is approximately 190 kb in length and located on chromosome 2q33. It consists of 13 exons and 12 introns, and its cDNA is approximately 4 kb long [3]. The *BMPR2* gene is a member of the transforming growth factor B (TGF-β) receptor superfamily (an important factor involved in embryonic development, angiogenesis, cell proliferation, tracheal and skeletal formation, and apoptosis), and it encodes a transmembrane serine-threonine kinase receptor; exons 1-3 encode the cell extracellular ligand binding domain, exon 4 encodes the transmembrane domain, exons 5-11 encode the intracellular serine–threonine structural region, and exons 12 and 13 encode the cytoplasmic tail region [4–6]. TGF-β signaling pathways include BMPR1 and BMPR2 receptor proteins, ligand bone morphogenetic proteins (BMPs), and the receptor substrate SMAD protein [7]. After activation of BMPs, the auxiliary receptor endoglin promotes the binding of BMPR1 and BMPR2 to BMPs, further phosphorylating the SMAD protein. The activated SMAD protein enters the nucleus and binds to cellular regulatory elements to regulate the transcription and expression of the target gene, thereby maintaining the morphology of blood vessels [8]. The signal transduction pathway can inhibit the proliferation and migration of cells in the vascular smooth muscle and pulmonary artery, and promote the differentiation and apoptosis of vascular cells [9]. Studies have shown that mutations in the *BMPR2* gene can affect SMAD channels, cause SMAD phosphorylation defects, and lead to loss function in the TGF-β signaling, thereby inducing proliferation of vascular smooth muscle [10]. At the same time, mutation of the *BMPR2* gene can affect the expression of BMPR2 protein with no mature or non-functionality of the *Endoglin* gene, leading to the loss of downstream kinase activity of BMPR2, which blocks the downstream channel and induces the occurrence of reactive pulmonary hypertension (PTH) [11]. The BMP signaling pathway not only is involved in maintaining normal morphology of blood vessels, but can also mediate pro-inflammatory responses and corresponding vascular endothelial changes. This is accomplished by down-regulating the Toll-like receptor signaling pathway, thus preventing the mutated *BMPR2* gene form acting on TGF-β channels to promote the release of pro-inflammatory mediators.

The *ACVRL1* gene is located on chromosomes 12q11∼q14. Its mutation types are diverse, including missense mutations, frameshift mutations caused by insertion or deletion mutations, nonsense mutations, and splice-site mutations, with insertion or deletion mutations as those with the largest proportion [12]. Exons 10 and 8 at the mutation sites are exons with a relatively large mutation of the *ACVRL1* gene, and the region in exon 10 called the NANDOR box (codon 479–489) is a high-risk region of *AVCRL1* mutation [13]. The NANDOR box is thought to play an important role in the regulation of TGF-β signaling transduction. Gene mutations in this region affect the regulation of the TGF-β pathway, which in turn leads to PTH characterized by endothelial and smooth muscle cell dysfunction and proliferation [14].

In summary, it is speculated that the *BMPR2* gene can mediate the inflammatory response and maintain normal structure of lung tissue, but whether the *BMPR2* gene is involved in the pathogenesis of hypertension is still unclear. Mutations in the *ACVRL1* gene affect the regulation of the TGF-β pathway and development of PTH. Smad9 is a novel transcriptional regulator of BMP signaling, and this mutation has an important effect on vascular morphology and vascular endothelial changes.

Taken together, the *BMPR2*, *ACVRL1*, and *SMAD9* genes may be involved in the occurrence of PTH, especially in the regulation of related signaling pathways. However, whether these genes affect the occurrence of hypertension is unclear. Common gene mutation sites were analyzed to investigate the the association between single nucleotide polymorphisms (SNPs) of the *BMPR2, ACVRL1*, and *SMAD9* genes with hypertension risk.

## Materials and methods

### General information

The study was conducted in 460 patients with essential hypertension (EH) (case group) and 460 age-matched healthy volunteers (±5) of the case group. The patients in the case group were 38–75 years old, and those in the control group were 40–73 years old. The subjects were included in the period from August 2014 to August 2017. Hypertension was defined as the mean value of three measurements per day showing systolic blood pressure ≥140 mmHg and sustained diastolic blood pressure (DBP) ≥90 mmHg, or patients undergoing antihypertensive therapy. Patients with secondary hypertension, diabetes, heart, liver, kidney, and other functional impairments or cardiovascular and cerebrovascular diseases were excluded. The control group had SBP <130 mmHg and DBP <80 mmHg, excluding patients with a history of diabetes, heart, liver and kidney damage, as well as other cardiovascular and cerebrovascular diseases. Clinical and demographic data were collected from all subjects, including age, gender, BMI, family history of hypertension, smoking history, and drinking history. All subjects signed informed consent and the study was approved by the hospital’s ethics committee.

### Genotype analysis

**Figure 1:**
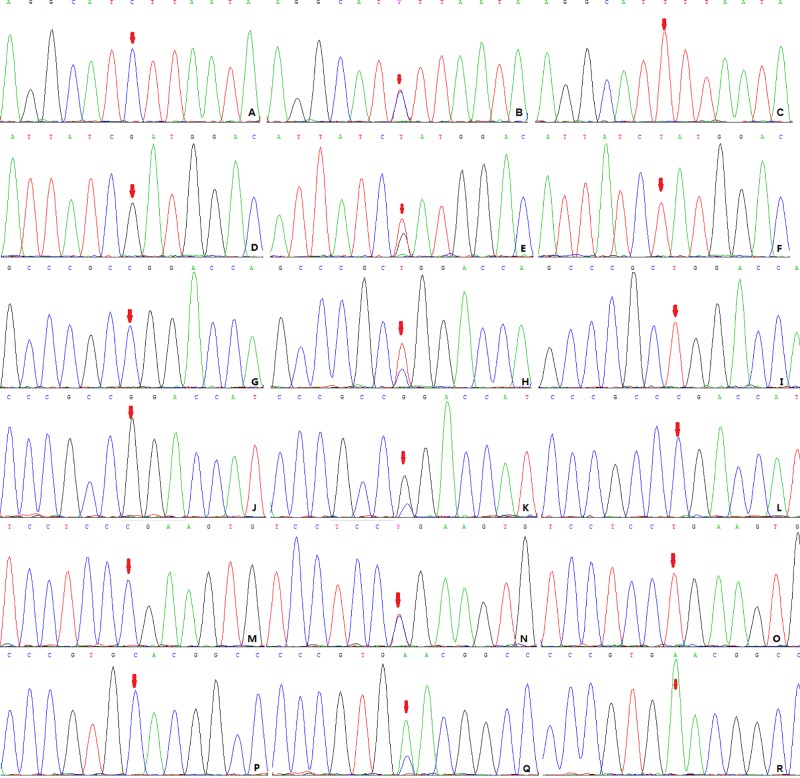
SNP site sequencing results of the *BMPR2*, *ACVRL1*, and *SMAD9* genes (**A–C**) is the CC, CT, and TT genotypes of the rs6435156 locus of the *BMPR2* gene; (**D–F**) is the GG, GT, and TT genotypes of the *BMPR2* gene rs1048829 locus. (**G–I**) shows the CC, CT, TT genotypes of the *ACVRL1* gene rs121909287. (**J–L**) shows the GG, GC, and CC genotypes of the rs121909284 locus of the *ACVRL1* gene. (**M–O**) shows the CC, CT, and TT genotypes of the *SMAD9* gene rs397514716 locus. (**P–R**) shows the CC, CA, and AA genotypes of the *SMAD9* gene rs121918359. The red arrow indicates the site of the mutation.

Blood sample (2 ml) in EDTA as anticoagulant was collected, leukocyte genomic DNA was extracted using the QIA amp DSP DNA Blood Mini Kit (Qiagen, Duesseldorf, German). A polymerase chain reaction (PCR) method was used to amplify the nucleotide fragments containing the target sequence using the extracted genomic DNA as a template. The PCR kit article number was SK2491-50 (Sangon Biotech Shanghai Co., Ltd, Shanghai, China). Primer was amplified by PCR according to the SNP site of the gene sequence information provided in Table 1. The PCR amplification conditions were as follows: an initial denaturation at 95°C for 5 min, followed by 30 cycles of denaturation at 95°C for 30 s, annealing at 60°C for 20 s, extension at 72°C for 20 s, final extension at 72°C for 5 min, and storage at 4°C. After PCR amplification, the target fragment was purified using a DNA Purification Kit (D0033, Beyotime, Shanghai, China). The genotype of the target fragment was then detected by Sanger sequencing (Figure 1).

**Table 1:** SNP site and PCR amplification primers

Polymorphism	Primer sequence (5′ to 3′)
*BMPR2* rs6435156	F: 5′-CCG CTC GAG TCA CAT TGT CAA ACA GAA TTT TTC-3′;
	R: 5′-ATT TGC GGC CGC AAA GTC ACC AGT CTT TGC TTG G-3′
*BMPR2* rs1048829	F: 5′-CCG CTC GAG ATC GAG AGT TAA GAT GTT TCT ATT TGA-3′;
	R: 5′-ATT TGC GGC CGC TGG GTT TCA AGT TGT TTT AAA AAT G-3′
*ACVRL1* rs121909287	F: 5′-ACT GAC ATC TGG GCC TTT GG-3′;
	R: 5′- TAC CCT GTG TAG GGT GGG C-3′
*ACVRL1* rs121909284	F: 5′-CAC GGA CTG CTT TGA GTC CT-3′;
	R: 5′-CCA CAG GCT GAT TCC CCT TT-3′
*SMAD9* rs397514716	F: 5′-AGG GTC GGT GAA CCC ATC TA-3′;
	R: 5′-TGA ACA ACC GAG TTG GGG AG-3′
*SMAD9* rs121918359	R: 5′- TCC CGT ATT TCC CCA CAG AC-3′
	F: 5′-CAC ACA ACG CCA CCT ATC CT-3′;

### Biochemical indicator testing

All subjects provided a 5-ml blood sample after fasting for 14 to 16 h. Biochemical analysis was performed at the hospital’s central laboratory. The blood samples were allowed to stand for half an hour and then centrifuged at 3500 × ***g***, followed by quantitative detection of triglycerides, HDL cholesterol, and LDL cholesterol by enzymatic techniques using a Hitachi 911 automated analyzer (Boehringer Mannheim BV, Almere).

### Statistical analysis

To determine whether the SNP site gene frequencies of the *BMPR2*, *ACVRL1*, and *SMAD9* genes were consistent with Hardy-Weinberg equilibrium, the χ2 testing was used. General demographic characteristics between the case and control groups were compared. The categorical variables were analyzed by χ2 test, and the continuous variables were analyzed by Student’s *t*-test or Mann–Whitney *U-*test. The odds ratio (OR) and the corresponding 95% confidence interval (CI) were determined. The positive Wald method was used to analyze the correlation between SNPs of the *BMPR2*, *ACVRL1*, and *SMAD9* genes and EH risk using a multivariate logistic regression model, and to adjust the age, gender, BMI, family history of hypertension, smoking history, drinking history, and other factors. Gene–environment interaction analysis was performed using multi-factor dimensionality reduction (MDR) (http://www.multifactordimensionalityreduction.org/), and haploid analysis was performed using the Haploview 4.2 software. SPSS 20.0 (IBM, Armonk, NY) was used for statistical analysis; all tests were two-tailed, and *P*<0.05 indicated that a difference was statistically significant.

## Results

### Clinical and demographic characteristics of the study population

Table 2 summarizes the clinical and demographic characteristics of the subjects enrolled in this study. The results showed that the proportion of patients with EH who also had a family history of hypertension was significantly higher than that of the control group. SBP and DBP were higher than the control group, and the difference was statistically significant (*P*<0.001). There was no significant difference in age, gender, BMI, smoking, alcohol consumption, triglyceride, HDL cholesterol, or LDL cholesterol between the case and control groups (*P*>0.05).

**Table 2:** Comparisons of general data in the case group and the control group

Parameters	Case (*n*=460)	Control (*n*=460)	*P* value
Age (years, mean ± SD)	51.7 ± 7.6	52.4 ± 8.0	0.174
Gender [*n* (%)]			
Male	290 (63.0%)	278 (60.4%)	0.420
Female	170 (37.0%)	182 (39.6%)	
Body mass index (kg/m^2^, mean ± SD)	22.8 ± 2.6	22.6 ± 2.5	0.235
Family history of hypertension [*n* (%)]			
Yes	193 (42.0%)	32 (7.0%)	<0.001
No	267 (58.0%)	428 (93.0%)	
Systolic blood pressure (mmHg, mean ± SD)	138.4 ± 15.3	116.1 ± 18.9	<0.001
Diastolic blood pressure (mmHg, mean ± SD)	87.7 ± 8.7	71.0 ± 8.8	<0.001
Smoking [*n* (%)]			
Yes	52 (11.3%)	55 (12.0%)	0.760
No	408 (88.7%)	405 (88.0%)	
Alcohol consumption [n (%)]			
Yes	78 (17.0%)	85 (12.0%)	0.550
No	382 (83.0%)	375 (81.5%)	
Triglyceride (mg/dl, mean ± SD)	168.2 ± 20.9	165.8 ± 20.2	0.077
HDL cholesterol (mg/dl, mean ± SD)	43.4 ± 9.4	43.0 ± 8.8	0.505
LDL cholesterol (mg/dl, mean ± SD)	89.9 ± 11.6	91.3 ± 13.5	0.092

Note: Corrected factors include age, gender, BMI, family history of hypertension, smoking history, and drinking history.Abbreviations: CI, confidence interval; HDL, high-density lipoprotein; LDL, low-density lipoprotein; OR, odds ratio.

### *BMPR2*, *ACVRL1*, and *SMAD9* gene polymorphisms and risk of EH

The *BMPR2* loci rs6435156 and rs1048829, the *ACVRL1* loci rs121909287 and rs121909284, and the *SMAD9* gene rs397514716 and rs121918359 loci in the study population were consistent with Hardy–Weinberg equilibrium (*P*>0.05). The multivariate logistic regression model showed that after adjusting for confounding factors (such as age, gender, BMI, family history of hypertension, smoking status, and drinking status), the rs6435156 and rs1048829 loci of the *BMPR2* gene, the *ACVRL1* gene rs121909287 locus, and the *SMAD9* gene rs397514716 locus were associated with EH risk. There was increased risk of EH in the *BMPR2* gene rs6435156 locus dominant (adjusted OR = 1.572, 95% CI: 1.385–1.765, *P*<0.001) and recessive (adjusted OR = 1.926, 95% CI: 1.693–2.067, *P*<0.001) models. The risk of EH was elevated in the recessive model of the rs1048829 locus of the *BMPR2* gene (adjusted OR = 1.444, 95% CI: 1.142–1.696, *P*=0.003), and there was no significant change in EH risk in the dominant model (*P*=0.465). EH risk in the *ACVRL1* gene rs121909287 locus recessive model was elevated (adjusted OR = 1.403, 95% CI: 1.101–1.660, *P*=0.008). EH risks in the *SMAD9* gene rs397514716 locus dominant (adjusted OR = 1.370, 95% CI: 1.183–1.559, *P*<0.001) and recessive (adjusted OR = 1.803, 95% CI: 1.470–1.983, *P*<0.001) models both increased. The *ACVRL1* gene rs121909284 locus and the *SMAD9* gene rs121918359 locus were not associated with EH risk (*P*>0.05) (Table 3).

**Table 3:** Correlation between the *BMPR2*, *ACVRL1*, and *SMAD9* gene polymorphisms and risk of EH.

SNP	Case (*n*=460)	Control (*n*=460)	*P* value	crude OR (95% CI)	*P* value	Adjusted OR (95% CI)
*BMPR2*						
rs6435156						
CC	287 (62.4%)	378 (82.2%)		1.00		
CT	105 (22.8%)	74 (16.1%)	<0.001	1.869 (1.319–2.649)	< 0.001	1.359 (1.154–1.574)
TT	68 (14.8%)	8 (1.7%)	<0.001	11.195 (5.097–25.594)	< 0.001	2.073 (1.813–2.233)
CT+TT	173 (37.6%)	82 (17.8%)	<0.001	2.779 (2.027–3.812)	< 0.001	1.572 (1.385–1.765)
CC+CT	392 (85.2%)	452 (98.3%)		1.00		
TT	68 (14.8%)	8 (1.7%)	<0.001	9.801 (4.482–22.318)	< 0.001	1.926 (1.693–2.067)
C	679 (73.8%)	830 (90.2%)		1.00		
T	241 (26.2%)	90 (9.8%)	<0.001	3.273 (2.497–4.294)	< 0.001	1.618 (1.478–1.752)
*BMPR2*						
rs1048829						
GG	324 (70.4%)	335 (72.8%)		1.00		
GT	98 (21.3%)	109 (23.7%)	0.647	0.930 (0.672–1.286)	0.706	0.963 (0.807–1.133)
TT	38 (8.3%)	16 (3.5%)	0.003	2.456 (1.296–4.699)	0.004	1.431 (1.128–1.687)
GT+TT	136 (29.6%)	125 (27.2%)	0.421	1.125 (0.836–1.514)	0.465	1.060 (0.913–1.219)
GG+GT	422 (91.7%)	444 (96.5%)		1.00		
TT	38 (8.3%)	16 (3.5%)	0.002	2.499 (1.327–4.756)	0.003	1.444 (1.142–1.696)
G	746 (81.1%)	779 (84.7%)		1.00		
T	174 (18.9%)	141 (15.3%)	0.041	1.289 (1.003–1.657)	0.048	1.129 (1.001–1.260)
*ACVRL1*						
rs121909287						
CC	291 (63.3%)	298 (64.8%)		1.00		
CT	132 (28.7%)	145 (31.5%)	0.630	0.932 (0.693–1.253)	0.683	0.965 (0.824–1.120)
TT	37 (8.0%)	17 (3.7%)	0.007	2.229 (1.185–4.228)	0.011	1.387 (1.083–1.651)
CT+TT	169 (36.7%)	162 (35.2%)	0.631	1.068 (0.809–1.411)	0.680	1.033 (0.898–1.183)
CC+CT	423 (92.0%)	443 (96.3%)		1.00		
TT	37 (8.0%)	17 (3.7%)	0.005	2.279 (1.222–4.289)	0.008	1.403 (1.101–1.660)
C	714 (77.6%)	741 (80.5%)		1.00		
T	206 (22.4%)	179 (19.5%)	0.122	1.194 (0.948–1.5050	0.136	1.090 (0.973–1.212)
*ACVRL1*						
rs121909284						
GG	287 (62.4%)	307 (66.7%)		1.00		
GC	142 (30.9%)	130 (28.3%)	0.288	1.168 (0.868–1.574)	0.323	1.080 (0.930–1.245)
CC	31 (6.7%)	23 (5.0%)	0.201	1.442 (0.794–2.627)	0.255	1.188 (0.884–1.486)
GC+CC	173 (37.6%)	153 (33.3%)	0.168	1.210 (0.914–1.600)	0.190	1.098 (0.956–1.255)
GG+GC	429 (93.3%)	437 (95.0%)		1.00		
CC	31 (6.7%)	23 (5.0%)	0.262	1.37 3(0.762–2.480)	0.326	1.159 (0.866–1.441)
G	716 (77.8%)	744 (80.9%)		1.00		
C	204 (22.2%)	176 (19.1%)	0.107	1.204 (0.954–1.520)	0.120	1.095 (0.977–1.217)
*SMAD9*						
rs397514716						
CC	345 (75.0%)	395 (85.9%)		1.00		
CT	81 (17.6%)	60 (13.0%)	0.018	1.546 (1.058–2.260)	0.023	1.232 (1.029–1.440)
TT	34 (7.4%)	5 (1.1%)	<0.001	7.786 (2.868–22.908)	<0.001	1.870 (1.521–2.060)
CT+TT	115 (25.0%)	65 (14.1%)	<0.001	2.026 (1.427–2.877)	<0.001	1.370 (1.183–1.559)
CC+CT	426 (92.6%)	455 (98.9%)		1.00		
TT	34 (7.4%)	5 (1.1%)	<0.001	7.263 (2.682–21.326)	<0.001	1.803 (1.470–1.983)
C	771 (83.8%)	850 (92.4%)		1.00		
T	149 (16.2%)	70 (7.6%)	<0.001	2.347 (1.720–3.205)	<0.001	1.430 (1.275–1.578)
*SMAD9*						
rs121918359						
CC	332 (72.2%)	345 (75.0%)		1.00		
CA	112 (24.3%)	101 (22.0%)	0.367	1.152 (0.837–1.587)	0.410	1.072 (0.913–1.243)
AA	16 (3.5%)	14 (3.0%)	0.645	1.188 (0.540–2.619)	0.784	1.088 (0.701–1.470)
CA+AA	128 (27.8%)	115 (25.0%)	0.331	1.157 (0.853–1.568)	0.370	1.074 (0.922–1.237)
CC+CA	444 (96.5%)	446 (97.0%)		1.00		
AA	16 (3.5%)	14 (3.0%)	0.710	1.148 (0.525–2.520)	0.853	1.069 (0.690–1.441)
C	776 (84.3%)	791 (86.0%)		1.00		
A	144 (15.7%)	129 (14.0%)	0.325	1.138 (0.872–1.484)	0.359	1.065 (0.933–1.202)

Note: Corrected factors include age, gender, BMI, family history of hypertension, smoking history, and drinking history. Abbreviations: CI, confidence interval; OR, odds ratio.

### Haplotype analysis

**Figure 2:**
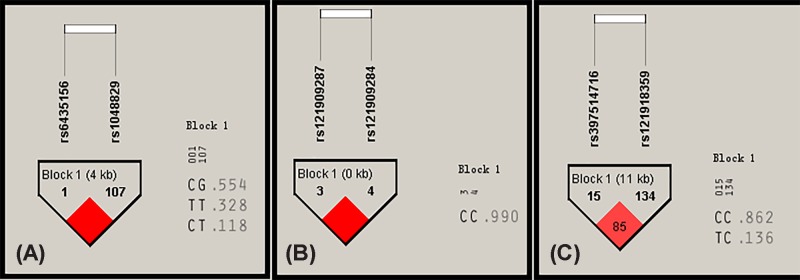
Linkage disequilibrium analysis of SNP loci in the *BMPR2*, *ACVRL1*, and *SMAD9* genes According to the human genomic haplotype map of the Chinese Han population in Beijing, Haploview 4.2 software was used to analyze the linkage disequilibrium of two SNPs of the *BMPR2*, *ACVRL1*, and *SMAD9* genes according to the human genome haplotype database. (**A**) is a linkage disequilibrium analysis of the rs6435156 and rs1048829 loci of the *BMPR2* gene. (**B**) shows the linkage disequilibrium analysis of the *ACVRL1* gene rs121909287 and rs121909284 loci. (**C**) shows the linkage disequilibrium analysis of the *SMAD9* gene rs397514716 and rs121918359 loci.

The Haploview 4.2 software was used to analyze linkage disequilibrium in the *BMPR2* gene rs6435156 and rs1048829 loci, the *ACVRL1* gene rs121909287 and rs121909284 loci, and the *SMAD9* gene rs397514716 and rs121918359 loci. Six haplotypes were constructed (Figure 2). Multivariate logistic regression models showed that after adjusting for confounding factors (such as age, gender, BMI, family history of hypertension, smoking status, and drinking status), the CG haplotypes (adjusted OR = 0.468, 95% CI: 0.390–0.557, *P*<0.001) constructed by the rs6435156 and rs1048829 loci of the *BMPR2* gene, CC haplotypes (adjusted OR = 0.839, 95% CI: 0.712–0.979, *P*=0.024) constructed by the rs121909287 and rs121909284 loci of the *ACVRL1* gene, and CC haplotype constructed by the *SMAD9* gene rs397514716 and rs121918359 loci (adjusted OR = 0.849, 95% CI: 0.730–0.982, *P*=0.026) were associated with decreased risk of EH. However, the TT haplotype constructed by the rs6435156 and rs1048829 loci of the *BMPR2* gene was associated with increased risk of EH (adjusted OR = 1.469, 95% CI: 1.291–1.658, *P*<0.001). The CT haplotype of the *BMPR2* gene rs6435156 and rs1048829 loci and the TC haplotype of the *SMAD9* gene rs397514716 and rs121918359 loci were not associated with EH risk (*P*>0.05) (Table 4).

**Table 4:** *BMPR2*, *ACVRL1*, and *SMAD9* gene haplotypes and EH Risk

Gene	Haplotype	Case (*n*=460)	Control (*n*=460)	OR (95% CI)	*P* value	Adjusted OR (95% CI)	Adjusted *P* value
*BMPR2*	CG	0.215	0.523	0.249 (0.185–0.336)	<0.001	0.468 (0.390–0.557)	<0.001
	TT	0.412	0.235	2.294 (1.291–1.658)	<0.001	1.469 (1.291–1.658)	<0.001
	CT	0.354	0.401	0.823 (0.625–1.085)	0.153	0.906 (0.785–1.041)	0.174
*ACVRL1*	CC	0.254	0.324	0.712 (0.529–0.958)	0.020	0.839 (0.712–0.979)	0.024
*SMAD9*	CC	0.308	0.381	0.727 (0.548–0.965)	0.022	0.849 (0.730–0.982)	0.026
	TC	0.326	0.343	0.925 (0.697–1.228)	0.576	0.961 (0.830–1.106)	0.625

Abbreviations: Acc, accuracy; Bac, balanced; CI, confidence interval; CV, cross-validation; OR, odds ratio.

### The impact of gene–environment interactions on EH risk

**Figure 3:**
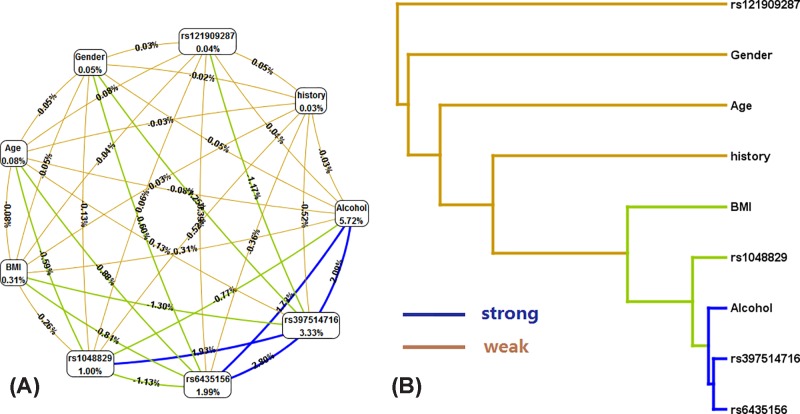
Effect of the *BMPR2*, *ACVRL1*, and *SMAD9* genes and environment interactions on EH risk The tree diagram and the ring diagram show blue to indicate strong interactions between the points, and the numerical value on the ring diagram indicates the correlation.

**Figure 4:**
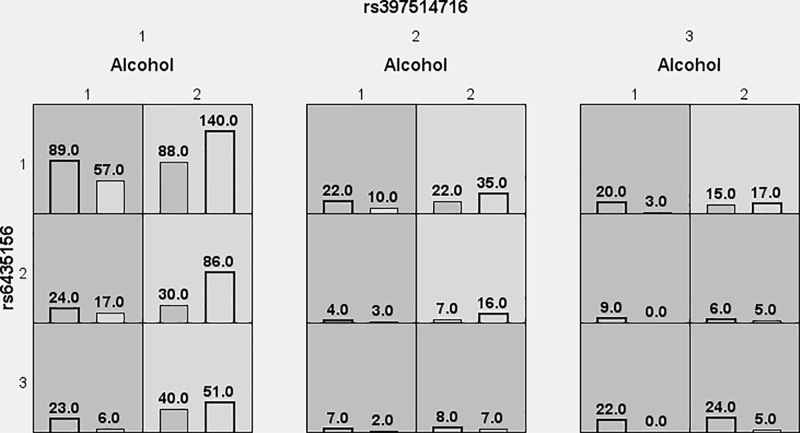
Graphical models of the gene–environment interactions The dark gray and light gray boxes represent a combination of high-risk and low-risk factors, respectively. The left column in each box represents the case group and the right column represents the control group. The height of the bar is proportional to the total number of samples in each group. Note that the patterns of high-risk and low-risk cells differ across each of the different multi-locus dimensions.“1” on the left side indicates the TT genotype of the rs6435156 locus of the *BMPR2* gene, “2” indicates the CT genotype, and “3” indicates the CC genotype. The horizontal uppermost layer “1” indicates the TT genotype of the *SMD9* gene rs397514716 locus, “2” indicates the CT genotype, and “3” indicates the CC genotype. The horizontal lower level “1” indicates a history of drinking, and the “2” indicates no drinking history.

The effect of SNPs of the *BMPR2*, *ACVRL1*, and *SMAD9* gene loci and environmental interactions on EH risk was analyzed by MDR (Figure 3). The results showed that the best models were alcohol, the rs6435156 locus of the *BMPR2* gene, and the rs397514716 locus of the *SMAD9* gene. The cross-validation consistency was 10/10, and the accuracy of the test sample was 0.678, *P*<0.001. The gene–environment interactions dendrogram and loop diagram showed strong interactions between the rs6435156 locus of the *BMPR2* gene and the rs397514716 locus of the *SMAD9* gene (Table 5). Further analysis showed that people with the TT genotype of the *BMPR2* gene rs6435156 locus, TT genotype of the *SMAD9* gene rs397514716 locus, and drinking history had the highest EH risk (OR = 4.523, 95% CI: 2.235–6.871, *P*<0.001) (Figure 4).

**Table 5:** Gene–environment interactions and EH risk model analysis results

Model	Bal. Acc. CV Training	Bal. Acc. CV Testing	CV consisitency	χ2 value	*P* value	OR (95% CI)
Alcohol	0.675	0.671	7/10	137.251	<0.001	9.668 (6.315–14.803)
Alcohol history	0.675	0.670	6/10	131.321	<0.001	9.542 (6.102–12.548)
Alcohol, rs6435156, rs397514716	0.678	0.687	10/10	132.444	<0.001	8.088 (5.481–11.934)
BMI, alcohol, rs6435156, rs397514716	0.618	0.620	6/10	130.69	<0.001	7.284 (5.050–10.506)
Gender, BMI, alcohol, rs6435156, rs397514716	0.585	0.591	5/10	121.011	<0.001	6.658 (4.718–9.396)
Gender, BMI, alcohol, rs6435156, rs121909287, rs397514716	0.594	0.549	4/10	120.017	<0.001	5.699 (4.177–7.775)
Gender, BMI, alcohol, age, rs6435156, rs1048829, rs121909287	0.603	0.5033	4/10	127.882	<0.001	5.737 (4.244–7.755)
Gender, BMI, alcohol, age, rs6435156, rs1048829, rs121909287, rs397514716	0.611	0.4696	6/10	147.524	<0.001	6.061 (4.487–8.187)

Alcohol history is indicated by “1,” No alcohol history “2”; BMI: ≥ mean is indicated by “1,” < mean “2”; Gender: Male “1,” Female “2.” Age: ≥ mean is indicated by “1,” < mean “2”; Hypertension history: Yes is “1,” No is “2.”

## Discussion

The vast majority of adult hypertension is EH, which is a complex polygenetic disease. Environmental and genetic factors both play an important role in the occurrence of this disease. Genetic factors lead to elevated blood pressure through multiple minor-effect genes and their interaction with environmental factors.

Changes in arterial structure and function are important pathological bases of EH. The reduction or inactivation of vascular wall elasticity, increase of peripheral resistance associated with vascular endothelial cell injury and arterial remodeling are closely related to continuous increase of blood pressure. The specific mechanism is still unclear and further research is needed for clarification.

Bone morphogenetic protein receptor Type 2 (BMPR2) is a member of the transforming growth factor beta receptor superfamily of the trans-membrane serine/threonine kinase receptor, and it exhibits high expression levels in various tissues, including the pulmonary vasculature and airway epithelium [15]. Wang et al. [16] found that rs6435156 C > T and rs1048829 G > T in the 3′-untranslated region of the *BMPR2* gene are associated with chronic obstructive pulmonary disease (COPD) risk in the southern Chinese population. Moreover, old age, smoking, family history of cancer, and rs6435156 C > T and rs1048829 G > T interactions lead to increased risk of COPD. However, there is currently no evidence supporting the correlation between mutations in the two SNP loci of the *BMPR2* gene, rs6435156 C > T and rs1048829 G > T, and EH risk. The results of Wang et al. [16] showed that the *BMPR2* gene rs6435156 C > T and rs1048829 G > T SNP loci are associated with BMPR2 deficiency, whereas Davies et al. [17] found that BMPR2 defects result in increased production of pro-inflammatory cytokines in pulmonary artery smooth muscle cells in *BMPR2* heterozygous mutant mice. In addition, Li et al. [18] showed that BMP4 (a ligand of BMPR2) is involved in the control of LPS-induced inflammatory responses in lung epithelial cells. In general, BMPR2 may be a key anti-inflammatory factor in the lung, and there is an evidence that BMPR2 plays an important role in the development of PTH [19]. Therefore, we speculated that the *BMPR2* gene rs6435156 C > T and rs1048829 G > T site SNP loci may be associated with the occurrence of EH. This is also the main reason for selecting these two SNP loci in this study. The results of this study showed that the frequencies of the rs6435156 and rs1048829 loci mutant allele of the *BMPR2* gene were significantly higher in EH patients than in the control group. The risk of EH was elevated in both the dominant and recessive models of the *BMPR2* gene rs6435156 locus. The risk of EH in the rs1048829 locus recessive model of the *BMPR2* gene increased, and there was no significant change in EH risk in the dominant model. The results showed that the rs6435156 and rs1048829 loci SNPs of the *BMPR2* gene were associated with EH risk. According to the results of Wang et al. [16], we speculated that the rs6435156 and rs1048829 loci SNPs of the *BMPR2* gene are associated with BMPR2 deficiency, and that BMPR2 deficiency leads to inhibition of SMAD protein phosphorylation, affecting the activation of SMAD protein and binding of intracellular regulatory elements. It blocks the phosphorylation of SMAD protein to be blocked and promotes the differentiation and apoptosis of vascular cells [20].

ACVRL1 (also known as ALK1) consists of 503 amino acids and is a TGF-β type I receptor. When the ligand binds to the TGF-β receptor, the type II receptor is first activated and then phosphorylated to activate the type I receptor, thereby forming a receptor complex. The activated receptor complex activates the downstream SMAD to form an oligomer and enter the cell to regulate the transcription of the corresponding gene, which determines the properties of endothelial cells during angiogenesis [21–23]. Piao et al. [24] studied the *ACVRL1* gene c.676G > A (p.V226M), c.955G > C (p.G319R), c.1231C > T (p.R411W), and c.1450C > T (p .R484W); each of these four mutations significantly reduced the phosphorylation level of Smad1/5 and decreased the activity of luciferase reporter, which affects the bone morphogenetic protein 9 (BMP-9) pathway and the occurrence of PTH. Currently, there is little research on the relationship between *ACVRL1* gene polymorphisms and EH. In this study, the rs121909287 (c.1231C > T) and rs121909284 loci of the *ACVRL1* gene were selected for study. The results showed that SNP of the rs121909287 locus was associated with EH risk, whereas there was no correlation between SNP of the rs121909284 locus and EH risk; the risk of EH was elevated in the recessive model of the *ACVRL1* gene rs121909287 locus. The analysis revealed that the rs121909287 locus is located in exon 8 and studies have reported that mutations in this region are associated with PTH [25], which may be related to altered ALK1 function after mutation, especially in the serine/threonine kinase domain of ACVRL1. Multiple mutations were found to be associated with PTH [24], and whether mutations at the rs121909287 site affect the serine/threonine kinase domain of ACVRL1 needs to be further investigated. In addition, exon 8 is considered to be an important functional region in the TGF-β I receptor that mediates angiogenesis and repair. Therefore, genetic mutations in this region are likely to affect blood vessel growth and repair, which in turn affects the occurrence of hypertension.

SMAD9 (also known as SMAD8) is an intracellular medium of BMPs. In the TGF-β signal transduction pathway, BMPR1 and BMPR2 receptor proteins and ligand BMPs bind to activate the receptor substrate SMAD protein. The activated SMAD protein enters the nucleus and binds to cellular regulatory elements to regulate the transcription and expression of the target genes, thereby maintaining the morphology of blood vessel. Kylie et al. [26] found that c.606 C > A (p.C202X) mutation in the *SMAD9* gene is associated with PTH. In this study, we selected the rs397514716 and rs121918359 loci of the *SMAD9* gene (c.606 C > A). We found that SNP of the *SMAD9* gene rs397514716 locus is related to EH risk. The results of Kylie et al. showed that SNP of the rs121918359 locus in the *SMAD9* gene is associated with EH risk. The risk of EH in both the dominant and recessive models was higher. However, unlike in Kylie et al. [26], a correlation between SNP and EH risk in the rs121918359 locus was not found in our study. These differences may be related to ethnic differences. In addition, the influence of environmental factors in different regions may also result in phenotypic differences.

To analyze the effects of genetic factors and environmental factors on EH, we analyzed the interactions of these SNP loci with environmental factors, and the results showed that the best models were alcohol, the rs6435156 locus, and the rs397514716 locus, suggesting that the interaction between these factors was the strongest. The gene–environment interactions dendrogram and ring diagram showed strong interactions between the rs6435156 locus of the *BMPR2* gene and the rs397514716 locus of the *SMAD9* gene, suggesting that these two SNP loci may have additive effects on EH risk. In addition, we constructed haploids of these genes. The results showed that the CG haploid constructed by the rs6435156 and rs1048829 loci of the *BMPR2* gene, CC haploid constructed by the rs121909287 and rs121909284 loci of the *ACVRL1* gene, and CC haploid constructed by the *SMAD9* gene rs121918359 and rs397514716 loci were protective against EH, whereas the TT haploid constructed by the rs6435156 and rs1048829 loci of the *BMPR2* gene was a risk factor for EH. Therefore, combined gene detection is necessary to predict EH risk.

There are still many shortcomings in this study. Owing to technical limitations, this study lacks an explanation of the molecular mechanism of the *BMPR2*, *ACVRL1*, and *SMAD9* gene polymorphisms and EH risk. In addition, the small sample size of this study may magnify any study error and the sample size must be expanded in future studies.

## Conclusion

SNPs in the rs6435156 and rs1048829 loci of the *BMPR2* gene, the rs121909287 locus of the *ACVRL1* gene, and the rs397514716 locus of the *SMAD9* gene were associated with EH risk in the Chinese Han population. Mutations may lead to an increased risk of EH, and the effects of alcohol and rs6435156 and rs397514716 mutations may add to the EH risk.

## Abbreviations

Acc: accuracy
Bac: balanced
BMPR2: bone morphogenetic protein receptor type 2
COPD: chronic obstructive pulmonary disease
CI: confidence interval
CV: cross-validation
DBP: diastolic blood pressure
EH: essential hypertension
HDL: high-density lipoprotein
LDL: low-density lipoprotein
MDR: multi-factor dimensionality reduction
OR: odds ratio
PTH: pulmonary hypertension
SNP: single nucleotide polymorphism
